# Extending DerSimonian and Laird's methodology to perform network meta‐analyses with random inconsistency effects

**DOI:** 10.1002/sim.6752

**Published:** 2015-09-30

**Authors:** Dan Jackson, Martin Law, Jessica K. Barrett, Rebecca Turner, Julian P. T. Higgins, Georgia Salanti, Ian R. White

**Affiliations:** ^1^ MRC Biostatistics UnitCambridgeUK

**Keywords:** method of moments, mixed treatment comparisons, multiple treatments meta‐analysis, network meta‐analysis

## Abstract

Network meta‐analysis is becoming more popular as a way to compare multiple treatments simultaneously. Here, we develop a new estimation method for fitting models for network meta‐analysis with random inconsistency effects. This method is an extension of the procedure originally proposed by DerSimonian and Laird. Our methodology allows for inconsistency within the network. The proposed procedure is semi‐parametric, non‐iterative, fast and highly accessible to applied researchers. The methodology is found to perform satisfactorily in a simulation study provided that the sample size is large enough and the extent of the inconsistency is not very severe. We apply our approach to two real examples. © 2015 The Authors. *Statistics in Medicine* Published by John Wiley & Sons Ltd.

## 1 Introduction

Meta‐analysis is well established in medical statistics and now requires little introduction. However, more sophisticated methods for meta‐analysis have recently been suggested, and here, we develop a new estimation method for network meta‐analysis. In network meta‐analysis, multiple treatments are included in the analysis, using data from trials that compare at least two of these treatments. This enables us to compare all treatments of interest, including any that may not have been compared directly. Models for network meta‐analysis reduce to standard pairwise meta‐analysis models when only two treatments are included, so that network meta‐analysis is a natural extension of the more conventional methods. See Salanti 1 for a review of the methods that have been developed for network meta‐analysis and Li *et al*. 2, Song *et al*. 3 and Cipriani *et al*. 4 for accounts of the type of concerns that accompany the use of network meta‐analysis.

Perhaps the greatest threat to the validity of a network meta‐analysis is the possibility of what has been called ‘inconsistency’ or ‘incoherence’. The consistency assumption states, for example, that the relative effect of treatment *B* to treatment *A* plus the relative treatment effect of treatment *C* to treatment *B* equals the relative effect of treatment *C* to treatment *A*. This assumption is necessarily true within a three‐arm trial involving treatments *A*, *B* and *C*. However, if the three comparisons are made in separate sets of studies, then it may be that direct observations of the relative effect of treatment *C* to treatment *A* do not agree with the sum of relative effects involving *B*. An example of this situation is provided by our second example in Section 7.2. Veroniki *et al.*, 5 conclude from their investigation that ‘About one‐eighth of the networks were found to be inconsistent’. Our position is that large inconsistencies in the evidence network should strongly discourage the application of models for network meta‐analysis, but small amounts of inconsistency should be anticipated and accounted for in the model. The impact of the inconsistency and the between‐study heterogeneity variances can be quantified using *I* ^2^ statistics (Section 5.3).

Attempts should be made to explain and/or remove or reduce inconsistency when it is found. This could include strategies such as adjusting for covariates that explain the inconsistency or performing subgroup analyses. Here, we assume that such strategies to eliminate any inconsistencies have failed, where this failure is taken to mean that the evidence for inconsistency has not been removed. We suggest that our methods for modelling inconsistency should be considered in situations where the evidence for inconsistency either cannot be entirely removed or in situations where unadjusted inferences are desired for the entire population of studies of interest and where the extent of the inconsistency is not too severe; we recognise however that the notion of what constitutes ‘severe inconsistency’ is likely to differ from one meta‐analyst to the next.

Here, we use random effects for the inconsistency parameters to describe any inconsistency that may be evident in the network. This means that we conceptualise inconsistency as an additional source of variation, in a similar way as between‐study heterogeneity is conceptualised in conventional random effects meta‐analyses. We expect the debate concerning the use of random or fixed effects for the inconsistency parameters to continue. Our use of random effects is largely motivated by our desire to be able to interpret the basic parameters as average effects across all studies of all designs.

We use one variance parameter to describe the between‐study variance and another variance parameter to describe the inconsistency. An advantage of this relatively simple model is that it is easily interpretable and the use of just two unknown variance parameters assists with model identifiability. More sophisticated models should be considered in application when the data are sufficient to identify them. However, most meta‐analysis datasets involve relatively few studies, and we anticipate that our model will be both appropriate and adequate for the majority of applications; for example, in their empirical investigation, Nikolakopoulou *et al.*, 6 find that the median number of studies per comparison is 2 (interquartile range 1–4). However, in situations where there is sufficient replication within designs, it will often be desirable to use more complicated models for the between‐study heterogeneity. Our modelling approach assumes that the consistency equations apply to the average treatment effects across all designs and studies, and we conceptualise inconsistency as another source of variation.

The aim of this paper is to develop a new procedure to fit the model proposed by Jackson *et al.* for network meta‐analysis 7 where here we use normal approximations for the within‐study distributions. We will show that this new procedure is an extension of DerSimonian and Laird's method 8 for univariate random effects meta‐analysis. Extensions of DerSimonian and Laird's methodology have previously been proposed for multivariate meta‐analysis 9, 10, but these are not immediately useful for network meta‐analysis because they make different assumptions about the unknown variance components. Jackson's model is a relatively simple model for network meta‐analysis, which, because of common heterogeneity assumptions, includes two unknown variance parameters that represent the extent of the between‐study heterogeneity and the inconsistency across the network. We follow the method of DerSimonian and Laird by estimating these two variance components using the method of moments. Upon approximating the true variance components with their estimates, the analysis proceeds as a weighted regression where all weights are treated as known. Lumley 11 proposed a model with random inconsistency terms for a network of two‐arm trials. Lumley's model is equivalent to Jackson's model when all studies are two‐arm trials and normal approximations are used. Hence, the proposed approach also provides a way to fit Lumley's model using the method of moments.

The general modelling framework we use here follows that proposed by Jackson *et al.*, 7. The estimation procedures proposed in Sections 3 and 4 are entirely new, however, as are the ideas for making further inferences using the proposed estimation procedure (Section 5) and the simulation study (Section 6). We also examine a new and highly challenging example in Section 7. The novel ideas presented here are potentially important methodologically because they provide new semi‐parametric and non‐iterative estimation methods for network meta‐analysis. These ideas are also potentially important for applied work because, as a direct extension of DerSimonian and Laird's procedure, they provide a method to extend the very many univariate meta‐analyses that use this methodology. The previous implementation of the proposed model 7 instead made use of WinBUGS 12. In contrast to this, the new estimation procedure proposed here is conceptually very similar to the two‐stage approach 13, but we assume a more standard between‐study heterogeneity structure for network meta‐analysis, and we further estimate an inconsistency variance, so that the consistency assumption can be relaxed. Our proposed methods avoid any difficulties that might be associated with the previous WinBUGS implementation, which include the statistical expertise required to choose appropriate priors, good parameterisations to ensure good mixing, checking convergence diagnostics and dealing with Monte Carlo error.

Our estimation method is motivated by the recently proposed decomposition of the *Q* statistic for network meta‐analysis 14, 15, which is a statistic that is also used in the context of multivariate meta‐regression 16. The proposed method can be adapted to fit certain types of loop inconsistency models 17, and we will explain how this can be achieved in Section 4.3. Loop inconsistency models are intended to reflect the sources of inconsistency that might arise when closed loops in the network occur. By allowing each design to estimate different treatment effects, the design‐by‐treatment interaction model includes more inconsistency parameters than loop inconsistency models 18. To our knowledge, our suggestion in Section 4.3 in the succeeding text is the first proposal for fitting loop inconsistency models with random inconsistency effects within a frequentist framework. The application of standard three‐level models 19 to network meta‐analysis, where the within‐study, between‐study and inconsistency variations provide the three levels, also warrants further investigation so that a likelihood‐based network meta‐analysis using random inconsistency effects might be performed. We return to this issue in the discussion.

The rest of the paper is set out as follows. In Section 2, we describe the model, and in Section 3, we present our new estimation method. In Section 4, we discuss some useful special cases of our method, and in Section 5, we discuss ways to make further inferences from the fitted model. In Section 6, we perform a simulation study, and in Section 7, we apply our method to two examples. We conclude with a discussion in Section 8.

## 2 The model

It is possible to fit models for network meta‐analysis using either contrast‐based or arm‐based analyses 20, 21, 22, and we will use a contrast‐based analysis. The previous implementation of our modelling approach 7 allowed arm‐based analyses, but the methods developed in this paper do not allow this.

In a contrast‐based analysis, the outcome data are estimated treatment effects, such as mean differences or log odds ratios. Without loss of generality, we take treatment *A* as the reference treatment and refer to the other treatments as *B*, *C*, *D* and so on. We follow the convention of taking the design *d* as referring only to the set of treatments compared in a trial 7, 14, 15, 18, 23. For example, if the first design compares treatments *A* and *B* only, then *d* = 1 refers to two‐arm trials that compare these two treatments. The trials may have more than two treatment arms.

Our model is a special case of the one proposed by Jackson *et al.*, 7, where here we use normal within‐study approximations. Hence, our modelling framework has been described previously, but we present our model again here so that this paper is self‐contained. Our model for network meta‐analysis is

(1) $$Y_{\mathrm{di}}=\delta_{d}+B_{\mathrm{di}}+\Omega_{d}+\varepsilon_{\mathrm{di}}$$

where ***Y***_*di*_ is the vector of estimated treatment effects from the *i*th study of design *d* and all ***B***_*di*_, ***Ω***_*d*_ and ***ε***_*di*_ are assumed to be independent. The length of ***Y***_*di*_ is *c*_*d*_, where *c*_*d*_ is the number of treatments included in design *d* minus one. To conveniently take a set of estimated effects from studies of design *d* to be used in analysis, and so provide ***Y***_*di*_ in model (1), we choose a baseline treatment in design *d*. The entries of ***Y***_*di*_ are then the estimated treatment effects of the other treatments included in design *d* relative to this baseline treatment. It is most straightforward to choose the baseline treatments as the treatment in each design that first appears in the alphabet.

The four components that comprise model (1) have previously been described by Jackson *et al.*, 7, but we also provide details of these immediately in the succeeding text in the context of the less general case assumed here where normal within‐study approximations are used for the ***ε***_*di*_.

### 2.1 The average treatment effects

We define the average (across all studies and designs) treatment effects relative to the reference treatment *A* as *δ* ^*AB*^, *δ* ^*AC*^, *δ* ^*AD*^ and so on, where these *δ* parameters are referred to as the basic parameters. These parameters are of primary interest. There are *c* basic parameters, where *c* equals the number of treatments in the network meta‐analysis minus one.

The vector ***δ***_*d*_ in model (1) then contains appropriate linear combinations of these basic parameters so that ***δ***_*d*_ denotes the average treatment effect corresponding to the vector of effects ***Y***_*di*_. For example, if the first design contains treatments *A*, *B* and *E*, and treatment *A* is taken as the baseline treatment for this design, then ***δ***_1_=(*δ* ^*AB*^,*δ* ^*AE*^)^*T*^. As another example, if the second design contains treatments *C*, *D*, *E* and *G*, where this design provides only indirect information about the basic parameters, and treatment *C* is taken as the baseline treatment for this design, then ***δ***_2_=(*δ* ^*AD*^−*δ* ^*AC*^,*δ* ^*AE*^−*δ* ^*AC*^,*δ* ^*AG*^−*δ* ^*AC*^)^*T*^. This means that we do assume that the consistency equations apply to the *average* treatment effects across all designs and studies, but we still allow for inconsistencies in the network by including the random inconsistency effects ***Ω***_*d*_.

### 2.2 The between‐study heterogeneity

The term ***B***_*di*_ contains study by treatment interaction terms, which model the between‐study heterogeneity. We assume that

$$B_{\mathrm{di}}∼N\left(\text{0},\tau_{\beta }^{2}P_{c_{d}}\right)$$

where ${\text{P}}_{c_{d}}$ denotes a *c*_*d*_×*c*_*d*_ matrix with ones on the leading diagonal and halves elsewhere. This is a standard assumption, justified by the assumption that the heterogeneity variance is the same for all treatment comparisons for every study 20. Using **J**_*a*_ to denote the *a* × *a* matrix where all entries are one, and **I**_*a*_ to denote the *a* × *a* identity matrix, we can write ${\text{P}}_{c_{d}}=\left({\text{I}}_{c_{d}}+{\text{J}}_{c_{d}}\right)/2$, which is useful in computation and in Appendix C.

### 2.3 The inconsistency parameters

The term ***Ω***_*d*_ models the inconsistency; if any ***Ω***_*d*_≠**0**, then the designs provide different treatment effects and the network is inconsistent. We assume that

$$\Omega_{d}∼N\left(\text{0},\tau_{\omega }^{2}P_{c_{d}}\right)$$

This form of the inconsistency variance components is justified in a very similar way to the assumed form of between‐study heterogeneity variance components in the previous section. We assume that the inconsistency variance is the same for all treatment comparisons for every design 7. This distributional assumption is however less familiar than the one made in Section 2.2 and is partly motivated by the fact that it leads to an especially simple model. The inconsistency is therefore modelled using design‐specific random effects. Our definition of consistency is that the inconsistency variance, which we will denote as $\tau_{\omega }^{2}$, is equal to zero. Those who are not prepared to model inconsistency as an additional source of random variation are likely to prefer the use of fixed effects for the inconsistency parameters. Furthermore, the inconsistency in a given network might instead be attributed to a variety of other reasons 24, 25.

### 2.4 The within‐study variation

We use normal approximations for the within‐study distributions so that

$$\varepsilon_{\mathrm{di}}∼N(\text{0},S_{\mathrm{di}})$$

This means we assume that the ***ε***_*di*_ are multivariate normal with within‐study covariance matrix ***S***_*di*_. This type of normal approximation is standard in univariate and multivariate meta‐analyses; for example, normal approximations are often used for log odds ratios, risk differences and so on. Because the estimates of treatment effect from a multi‐arm study share a common baseline treatment group, the within‐study covariances (the non‐diagonal entries of ***S***_*di*_) are non‐zero, and it is important that these are not ignored in the analysis 26. We regard the ***S***_*di*_ as fixed and known in the network meta‐analysis.

Together, these assumptions provide the marginal distribution $Y_{\mathrm{di}}∼N\left(\delta_{d},\left(\tau_{\beta }^{2}+\tau_{\omega }^{2}\right)P_{c_{d}}+S_{\mathrm{di}}\right)$, but when presenting this marginal distribution, it is important to recognise that the ***Y***_*di*_ from the same design *d* are correlated because they share a common inconsistency random effect.

## 3 Estimation

The model described in Section 2 has (*c* + 2) parameters: the *c* basic parameters (*δ* ^*AB*^, *δ* ^*AC*^, *etc.*) that describe the average treatment effects relative to treatment *A* and two variance components $\tau_{\beta }^{2}$ and $\tau_{\omega }^{2}$ that describe the extent of the between‐study heterogeneity and the inconsistency, respectively. The estimation will proceed in the usual way when using the method of moments for meta‐analysis 8, 9, 10: we begin by estimating the variance components and then treating them as fixed and known. This is a justifiable assumption provided that both the number of designs, and the replication of studies within designs and between designs, are great enough to accurately estimate $\tau_{\omega }^{2}$ and $\tau_{\beta }^{2}$, respectively. Although this is often not the case in practice, the simulation study in Section 6 in the succeeding text suggests that networks with relatively little replication within and between designs provide reasonably accurate inferences.

### 3.1 The estimating equations

To estimate $\tau_{\beta }^{2}$ and $\tau_{\omega }^{2}$ using the method of moments, we require two estimating equations that are to be obtained from the expectation of quadratic forms 8, 9, 10. Appropriate quadratic forms for this purpose are given by the recently proposed decomposition of the *Q* statistic for network meta‐analysis 14, 15, which is given by

(2) $$Q^{\text{net}}=\overset{D}{\underset{d=1}{\sum }}Q_{d}^{\text{het}}+Q^{\text{inc}}$$

where *D* is the number of designs, *Q* ^net^ is the scalar Cochran‐type *Q* statistic for the entire network meta‐analysis (given as *Q*_*s*_ in equation (7) of Jackson *et al.*, 16, and as *Q* in equation (9) of Gasparrini *et al.*, 27 in the context of multivariate meta‐analysis) and $Q_{d}^{\text{het}}$ is this *Q* statistic calculated using only studies of the *d*th design; these *Q* statistics are defined in the succeeding text. Equation (2) provides three quadratic forms, but clearly these are linearly dependent, and two estimating equations follow from (2), as required to estimate $\tau_{\beta }^{2}$ and $\tau_{\omega }^{2}$. We will obtain estimating equations by matching *Q* ^net^ and $\sum \overset{\text{het}}{\underset{d}{Q}}$ to their expectations. The expectation of *Q* ^inc^ and a third, linearly dependent, estimating equation could then be obtained by subtraction.

Moment‐based proposals for estimating the between‐study variance $\tau_{\beta }^{2}$ have previously been proposed by Lu *et al.*, 13 and Rücker and Schwarzer 28. The point estimates of the variance components can be used to perform both point and (approximate) interval estimation for the treatment effect parameters, which are the parameters of primary interest.

#### 3.1.1 The first estimating equation

We will obtain our first estimating equation by calculating $E\left[Q_{d}^{\text{het}}\right]$ so that $E\left[\sum \overset{\text{het}}{\underset{d}{Q}}\right]$ is obtained by summation. No inconsistency between trials of the same design is possible under model (1). Hence, the variation in the estimates from the same design is assumed to be due to within‐study sampling variation and between‐study variation. An implication of this for our modelling framework is that no inconsistency terms should be included in the model for a subgroup analysis where only trials of the same design are included.

In order to define $Q_{d}^{\text{het}}$ and evaluate its expectation, we stack the ***Y***_*di*_ to form ***Y***_*d*_ and we use the design‐specific marginal model for this vector

(3) $$Y_{d}∼N\left(X_{d}\beta_{d},S_{d}+\tau_{\beta }^{2}{\text{I}}_{n_{d}}⊗{\text{P}}_{c_{d}}\right)$$

where ⊗ denotes the Kronecker product and *n*_*d*_ is the number of studies of design *d*. Because ${\text{I}}_{n_{d}}$ denotes the *n*_*d*_×*n*_*d*_ identity matrix, ${\text{I}}_{n_{d}}⊗{\text{P}}_{c_{d}}$ is a block diagonal matrix where all submatrices along the block diagonal are ${\text{P}}_{c_{d}}$. The ‘regression’ design matrix **X**_*d*_ is obtained by stacking *n*_*d*_ identity matrices ${\text{I}}_{c_{d}}$, and ***β***_*d*_=***δ***_*d*_+***Ω***_*d*_ is a vector containing the design‐specific treatment effects. Finally, **S**_*d*_ is the block diagonal square matrix containing the **S**_*di*_ matrices, which we denote as **S**_*d*_=diag(**S**_*di*_). The scalar $Q_{d}^{\text{het}}$ is then calculated as

(4) $$Q_{d}^{\text{het}}={\left(Y_{d}-{\hat{Y}}_{d}\right)}^{T}S_{d}^{-1}\left({Y_{d}-\hat{Y}}_{d}\right)=\overset{n_{d}}{\underset{i=1}{\sum }}{\left(Y_{\mathrm{di}}-{\hat{Y}}_{\mathrm{di}}\right)}^{T}S_{\mathrm{di}}^{-1}\left({Y_{\mathrm{di}}-\hat{Y}}_{\mathrm{di}}\right)$$

where ${\hat{Y}}_{\mathrm{di}}$ is the fitted value of **Y**_*di*_ under model (3) where $\tau_{\beta }^{2}=0$. Writing $W_{d}=S_{d}^{-1}$ and $B_{d}=W_{d}-W_{d}X_{d}{\left(X_{d}^{T}W_{d}X_{d}\right)}^{-1}X_{d}^{T}W_{d}$, in Appendix A, we show that

(5) $$E\left(Q_{d}^{\text{het}}\right)=(n_{d}-1)c_{d}+K_{d}\tau_{\beta }^{2}$$

where

(6) $$K_{d}=\text{tr}\left(B_{d}\left({\text{I}}_{n_{d}}⊗{\text{P}}_{c_{d}}\right)\right)$$

and tr(·) denotes the trace operator. Equation (5) shows that the expectation of the quadratic form $Q_{d}^{\text{het}}$ is equal to its associated degrees of freedom plus the product of a fixed constant and the heterogeneity variance, just as it is in the standard univariate case 8. Summing (5) across all designs gives

$$E\left[\overset{D}{\underset{d=1}{\sum }}Q_{d}^{\text{het}}\right]=\overset{D}{\underset{d=1}{\sum }}(n_{d}-1)c_{d}+\tau_{\beta }^{2}\overset{D}{\underset{d=1}{\sum }}K_{d}$$

Replacing $E\left[\sum Q^{\text{het}}\right]$ with its observed value and $\tau_{\beta }^{2}$ with ${\hat{\tau }}_{\beta }^{2}$, we obtain our first estimating equation:

(7) $$\overset{D}{\underset{d=1}{\sum }}Q_{d}^{\text{het}}=\overset{D}{\underset{d=1}{\sum }}(n_{d}-1)c_{d}+{\hat{\tau }}_{\beta }^{2}\overset{D}{\underset{d=1}{\sum }}K_{d}$$

where (6) can be used to evaluate *K*_*d*_. We propose solving (7) for ${\hat{\tau }}_{\beta }^{2}$ to produce an ‘untruncated’ (possibly negative) estimate ${\hat{\tau }}_{\beta }^{2}$.

#### 3.1.2 The second estimating equation

We will obtain our second estimating equation by calculating E[*Q* ^net^]. We now vertically stack all the **Y**_*d*_ to produce **Y** and use the marginal model implied by our model described in Section 2:

(8) $$Y∼N\left(X\delta ,S+\tau_{\beta }^{2}{\text{P}}_{1}+\tau_{\omega }^{2}{\text{P}}_{2}\right)$$

where **S** is the block diagonal matrix containing the **S**_*d*_ and so contains the **S**_*di*_ matrices. The vector ***δ*** contains the basic parameters, so that ***δ*** = (*δ* ^*AB*^,*δ* ^*AC*^,*δ* ^*AD*^,⋯)^*T*^ and the design matrix **X** provides the mean structure for **Y** described in Section 2.1; **X** is therefore not obtained by stacking the **X**_*d*_ matrices in Equation (3). The matrices **P**_1_ and **P**_2_ are square matrices of appropriate dimension that contain entries from the set {0,1/2,1} to provide the variance structures described in Sections 2.2 and 2.3, respectively. This means that **P**_1*ij*_=0 if the corresponding entries of **Y** in model (8), **Y**_*i*_ and **Y**_*j*_, are from separate studies; otherwise, **P**_1*ii*_=1 and **P**_1*ij*_=1/2 for *i* ≠ *j*. Similarly, **P**_2*ij*_=0 if the corresponding entries **Y**_*i*_ and **Y**_*j*_ are from different designs; otherwise, **P**_2*ij*_=1 if **Y**_*i*_ and **Y**_*j*_ relate to the same treatment effect and **P**_2*ij*_=1/2 if **Y**_*i*_ and **Y**_*j*_ relate to different treatment effects within the same design.

We have that

$$Q^{\text{net}}={\left(Y-\hat{Y}\right)}^{T}W(Y-\hat{Y}).$$

where ***W*** = ***S*** ^−1^ and $\hat{Y}$ is the fitted value of ***Y*** under model (8) with $\tau_{\beta }^{2}=\tau_{\omega }^{2}=0$. Defining ***B*** = ***W*** − ***W*** ***X***(***X*** ^*T*^ ***W*** ***X***)^−1^ ***X*** ^*T*^ ***W***, in Appendix B, we show that

(9) $$E[Q^{\text{net}}]=\left(\overset{D}{\underset{d=1}{\sum }}n_{d}c_{d}\right)-c+\tau_{\beta }^{2}\text{tr}({\text{BP}}_{1})+\tau_{\omega }^{2}\text{tr}({\text{BP}}_{2})$$

so that our second estimating equation is

(10) $$Q^{\text{net}}=\left(\overset{D}{\underset{d=1}{\sum }}n_{d}c_{d}\right)-c+{\hat{\tau }}_{\beta }^{2}\text{tr}({\text{BP}}_{1})+{\hat{\tau }}_{\omega }^{2}\text{tr}({\text{BP}}_{2})$$

We propose substituting the ‘untruncated’ estimate from (7) into (10) and solving for ${\hat{\tau }}_{\omega }^{2}$ to produce an ‘untruncated’ (again, possibly negative) estimate ${\hat{\tau }}_{\omega }^{2}$. Together, Equations (7) and (10) provide simultaneous equations, and hence, in practice, we estimate the two unknown variance components at the same time. By producing ‘untruncated’ estimates of the unknown variance components by matching moments in this way, these untruncated estimators are unbiased under the assumptions of the model.

### 3.2 Inference for the average treatment effects

As in the univariate case 8, the estimated variance components can be negative unless they are truncated to prevent this. In the event that either or both of them are negative, we propose following the univariate convention and truncating ${\hat{\tau }}_{\beta }^{2}$ and ${\hat{\tau }}_{\omega }^{2}$ to zero when making inferences about the treatment effects, which results in positive bias. We then follow the further convention of approximating the true variance components with their estimates when making inferences about the average treatment effects. We denote the total estimated variance of **Y** by

(11) $$V=S+{\hat{\tau }}_{\beta }^{2}{\text{P}}_{1}+{\hat{\tau }}_{\omega }^{2}{\text{P}}_{2}$$

Then the estimated basic parameters, which are the average treatment effects relative to treatment *A*, are

(12) $$\hat{\delta }={\left(X^{T}V^{-1}X\right)}^{-1}X^{T}V^{-1}Y$$

which is approximately multivariately normally distributed with covariance matrix

(13) $$\mathrm{Var}(\hat{\delta })={\left(X^{T}V^{-1}X\right)}^{-1}$$

so that confidence intervals and the results from hypothesis tests can be obtained.

The estimates of the basic parameters contained in $\hat{\delta }$ describe the average treatment effects relative to the reference treatment *A*. The average treatment effects relative to other treatments can be obtained as appropriate linear combinations of $\hat{\delta }$. The normality approximation for $\hat{\delta }$ results in straightforward inference for these linear combinations and hence for all average treatment effects estimable from the network meta‐analysis. The treatment effects are interpreted as average treatment effects across all studies of all designs.

## 4 Special cases of the method

The previous section describes our proposed method for the analysis of network meta‐analysis datasets. Our method estimates both the heterogeneity and the inconsistency variances but collapses to simpler models when one or both of these variances are zero. This is reminiscent of random effect models collapsing to a fixed effect model when the between‐study variance is zero. Some simplifications of our proposed method are therefore both immediate and useful. Although our primary proposal is to fit our full model, we recognise that others may prefer to use alternative models, and so we describe some variations that we anticipate other meta‐analysts are likely to find attractive. For example, the consistency model may be preferred when the consistency assumption is supported by the data and loop inconsistency models may be preferred when the inconsistency in the network is to be attributed to a particular closed loop or loops.

### 4.1 Inference under the consistency assumption

In our framework, the consistency assumption is equivalent to assuming that $\tau_{\omega }^{2}=0$. Hence, our method could very easily be modified to assume consistency by constraining ${\hat{\tau }}_{\omega }^{2}=0$ in Equations (11), (12) and (13). This is because (7) provides an estimate of ${\hat{\tau }}_{\beta }^{2}$ regardless of the value $\tau_{\omega }^{2}$. Furthermore, this ${\hat{\tau }}_{\beta }^{2}$ could be used for all values of ${\hat{\tau }}_{\omega }^{2}$ assumed in the context of a sensitivity analysis if the inconsistency variance is to be used as the sensitivity parameter.

However, the estimate of $\tau_{\beta }^{2}$ from (7) makes no use of the additional information contained in the consistency assumption. Furthermore, it appears strange that the estimate of one variance component should be unaffected by the assumed magnitude of another variance component in the model, for this is not the case when using likelihood‐based or Bayesian methods. The issue is whether the sum of squares due to inconsistency is ignored or attributed to heterogeneity: under the consistency model, either is correct, but the latter provides more precise estimation. Hence, for an analysis assuming consistency, we propose calculating ${\hat{\tau }}_{\beta }^{2}$ from Equation (10) with ${\hat{\tau }}_{\omega }^{2}$ constrained to be zero, truncating ${\hat{\tau }}_{\beta }^{2}$ to zero if necessary and then proceeding as shown in Equations (11), (12) and (13) with ${\hat{\tau }}_{\omega }^{2}$ set to zero. This does not result in the same estimation as Lu *et al.*, 13 because here we assume a different, and more conventional, structure for the between‐study variance components.

In a standard univariate meta‐analysis, a fitted random effects model ‘collapses’ to (gives the same point estimate and confidence interval) a fixed effect model whenever the estimated between‐study variance is zero. Fitted consistency models, as described in this section, collapse to a ‘common‐effect and consistent’ model ( $\tau_{\beta }^{2}=0$ and $\tau_{\omega }^{2}=0$) when ${\hat{\tau }}_{\beta }^{2}=0$. Furthermore, fitted full models collapse to a common‐effect and consistent model when ${\hat{\tau }}_{\beta }^{2}={\hat{\tau }}_{\omega }^{2}=0$. However, a fitted full model does not collapse to the corresponding fitted consistency model when ${\hat{\tau }}_{\omega }^{2}=0$ when using our proposal for fitting consistency models. If we instead used Equation (7) to estimate $\tau_{\beta }^{2}$ when assuming consistency, then fitted full models would collapse to fitted consistency models when ${\hat{\tau }}_{\omega }^{2}=0$.

### 4.2 The standard method of DerSimonian and Laird

In Appendix C, we show that DerSimonian and Laird's estimating equation for the between‐study heterogeneity variance is recovered as a special case when all studies are two‐arm trials comparing the same two treatments. No inconsistency terms are included when all studies are of the same design (Section 3.1.1). Hence, the proposed procedure is a direct extension of DerSimonian and Laird's method for network meta‐analysis as claimed in the title of the paper.

### 4.3 Loop inconsistency models

Loop inconsistency models contain a subset of the inconsistency parameters contained in the design‐by‐treatment interaction model 18, 23. Because the proposed model (1) is a special case of the design‐by‐treatment interaction model 7, loop inconsistency models with random inconsistency effects can be incorporated into the estimation procedure by modifying **P**_2_. For example, this may involve setting some further entries to zero (the entries that correspond to any inconsistency parameters in our model that are not included in the loop inconsistency model).

Estimation then proceeds as explained in the previous text but using this modified **P**_2_ matrix. Hence, the proposed methodology can also incorporate loop inconsistency models. Lu and Ades 17 suggest comparing the magnitudes of $\tau_{\beta }^{2}$ and $\tau_{\omega }^{2}$ in the context of loop inconsistency models, and this can also be performed when using the model presented here by taking the ratio of the point estimates.

## 5 Further inferences

It is not sufficient to make inferences about only the basic parameters because other aspects of the model fit are also important 7. In this section, we describe how to perform other inferences using the proposed estimation method, and alternative ideas when the proposed method is not so amenable.

### 5.1 Uncertainty in the variance components

A disadvantage of our proposed approach is that measures of uncertainty in the variance components are not immediate from the methods described in the previous text just as they were not in the original account of DerSimonian and Laird 8. The recently proposed methods for the interval estimation of moments‐based estimates of between‐study variance, developed for univariate random effects meta‐analysis 29 and meta‐regression 30, have the potential to be extended for use with the model proposed here. However, this type of extension is not immediate when studies with more than two treatment arms are included in the analysis because then some of the estimates are not independent, and here, we have two unknown variance components. This type of extension could form the subject of future work.

A practical alternative is to use some form of bootstrapping to obtain an indication of the uncertainty in the variance components. A variety of approaches to performing bootstrapping in meta‐analysis have been proposed 31, 32. These methods could be used to obtain standard errors and/or confidence intervals of either the truncated and/or the untruncated estimates of the variance components, and we can obtain these on the variance or the standard deviation scale as desired. However, the best form of bootstrapping for this type of purpose is a special investigation in its own right. For now, we will omit statements about the uncertainty in the variance components, as is quite common practice when implementing the more familiar DerSimonian and Laird procedure for random effects meta‐analysis, but we do not regard this as ideal. This comment also applies to the extensions of DerSimonian and Laird's method for multivariate (multiple outcomes) meta‐analysis 9, 10. The best way to allow for the uncertainty in the variance components when making inferences about the treatment effects is another important topic for further work.

### 5.2 Ranking of the treatments

The probabilistic ranking of the treatments is often an important aim in network meta‐analysis. For example, the intention might be that the results from the network meta‐analysis are to feed into a decision analysis. Probabilistic ranking is most naturally performed in a Bayesian framework, but here, we adopt a classical approach.

The probabilistic ranking of the treatments can be performed in a similar way to White *et al.*, 23. Here, we simulate treatment effect vectors from a normal distribution centred at $\hat{\delta }$ from Equation (12) and with covariance matrix given by $\mathrm{Var}(\hat{\delta })$ from Equation (13). We then record the proportion of simulated treatment effect vectors in which each treatment is the most effective, and this estimates the probability that each treatment is best. The proportion of simulated treatment effect vectors in which each treatment is the second most effective estimates the probability that each treatment is second best, and so on. It is important to consider the whole distribution of the rankings for each treatment; the probability of being the best can be a misleading guide when ranking the treatments, especially when there is little statistical information about the treatment network. In our first example (Section 7.1), we show the probabilities that each treatment effect is best so that our new results can be compared with those obtained previously 7. However, the parameter estimation provides the focus of this paper, and so we do not make any formal attempt to rank the treatments in either example in Section 7.

This proposed method for the probabilistic ranking of treatments takes the average of all studies and all designs as the target for inference. We could also rank the treatments in a new study, or a new design, using our model 7.

### 5.3 *I* ^2^ statistics

Because we have based our estimation procedure on *Q* statistics, it is tempting to calculate *I* ^2^ statistics as (*Q* − *v*)/*Q*, where *Q* is an appropriate *Q* statistic and *v* is its associated degrees of freedom, in the usual way 16, 33. However, the justification for calculating *I* ^2^ statistics using this idea requires an explanation of why the resulting expression estimates a suitable quantity 16, 33. It is not obvious that the *Q* statistics used here are capable of producing meaningful *I* ^2^ statistics in this way. We therefore suggest, if *I* ^2^ statistics that describe the impact of the between‐study heterogeneity and/or the inconsistency are desired, that the methods used by Jackson *et al.*, 7 for calculating *I* ^2^ statistics should also be used with the estimation procedure suggested here. So that this paper is self‐contained, we will also describe these methods in this section.

We explain how to fit the full model in Section 3, and we refer to this as the ‘random‐effects and inconsistent’ (RI) model, because both between‐study heterogeneity and inconsistency are incorporated into the model. We explain how to fit the model under the consistency assumption in Section 4.1, and we refer to this as the ‘random‐effects and consistency’ (RC) model. It is also straightforward to fit the model under the assumption that both $\tau_{\beta }^{2}$ and $\tau_{\omega }^{2}$ are zero; here, we use Equations (11) and (12) with **V** = **S**, and we refer to this as the ‘common‐effect and consistent’ (CC) model. We denote the volumes (or generalised notions of volume in more than three dimensions) of the 95% (or some other coverage probability, this coverage probability is immaterial) confidence regions for all *c* basic parameters from these three models as *V*_*CC*_, *V*_*RC*_ and *V*_*RI*_, and the covariance matrices of the estimates of the *c* basic parameters **C**_*CC*_, **C**_*RC*_ and **C**_*RI*_, respectively. The *R* statistic comparing a model with an additional source (or sources) of variation to an alternative, reduced model, is given by the *c*th root of the ratio of the volumes of the confidence regions resulting from these two models, where the model with additional variation appears in the numerator.

For example, the *R* statistic comparing the ‘random‐effects and inconsistent’ and the ‘random‐effects and consistent’ models, which describes the impact of including inconsistency in the random effects model, is given by

(14) $$R={\left(\frac{V_{\mathrm{RI}}}{V_{\mathrm{RC}}}\right)}^{1/c}={\left(\left|C_{\mathrm{RI}}C_{\mathrm{RC}}^{-1}\right|\right)}^{1/2c}$$

where |·| denotes the matrix determinant, and we then define *I* ^2^ statistics as *I* ^2^=(*R* ^2^−1)/*R* ^2^, where we multiply these *I* ^2^ statistics by 100% in order to express them as percentages. The *R* statistic comparing the ‘random‐effects and inconsistent’ and the ‘common‐effect and consistent’ models is obtained by replacing ‘RC’ with ‘CC’ in (14); similarly, the *R* statistic comparing the ‘random‐effects and consistent’ and the ‘common‐effect and consistent’ models is obtained by replacing ‘RI’ with ‘RC’ and ‘RC’ with ‘CC’, respectively.

If desired, we may also obtain *R*, and hence *I* ^2^, statistics for particular subsets of the basic parameters 16 rather than for all the basic parameters and hence the network meta‐analysis as a whole. For example, we could take **C**_*RI*_ and **C**_*RC*_ as the submatrices for a subset of basic parameters and replace *c* with the reduced dimension of either **C**_*RI*_ and **C**_*RC*_ when computing (14). This could be of particular interest when different types of treatments are included in the network, for example pharmacological and non‐pharmacological treatments, so that the impact of the heterogeneity on different types of treatment can be quantified. *I* ^2^ statistics can also be calculated for any linear combination of treatment effects that are estimable in the network meta‐analysis that might be of inferential interest, and not just for the treatment effects relative to treatment *A*, by using appropriate linear combinations 16.

An investigation into the use of *I* ^2^ statistics of the type (*Q* − *v*)/*Q* may form the subject of future work, but we suggest that they should not be used until their use is clearly and properly justified. We will also refrain from giving thresholds to interpret our *I* ^2^ statistics because meta‐analysts may not agree on how to interpret the magnitudes of standard univariate *I* ^2^ statistics. Despite this, whatever convention the analyst is comfortable in using when interpreting standard *I* ^2^ statistics can also be used when interpreting the magnitude of the *I* ^2^ statistics described in this section.

### 5.4 Model section

Jackson *et al*. 7 used deviance information criterion (DIC) statistics 34 to determine the model fit and to decide whether or not the extra variance parameter in the inconsistency model is needed to adequately describe the data. Here, we do not adopt a likelihood‐based approach and so have no such general method such as DIC or AIC to determine model fit. We leave the best way to assess the adequacy of the consistency model, compared with the full model, as further work, but for now, the approach of White *et al*. 23 is probably the best frequentist approach for assessing the strength of evidence against the consistency assumption. This approach provides a global hypothesis test for the presence of inconsistency using fixed effects for the inconsistency parameters.

### 5.5 Locating inconsistencies in the network

Jackson *et al.*, 7 used the posterior distributions of ***Ω***_*d*_ to identify where the inconsistencies in the network occur. This was convenient because WinBUGS was used to fit the model. However, the resulting inferences for the ***Ω***_*d*_ could be driven by normality assumptions, and although empirical Bayes estimates of the ***Ω***_*d*_ using our approach could be derived, this approach is not nearly as convenient when using the method of moments as proposed here.

Hence, we suggest that alternative methods are more suitable for identifying where the inconsistency arises. These include the fixed effect inconsistency parameters approach of White *et al.*, 23 and node splitting 35. The proposed approach is intended as a simple and direct way of performing pooling across a network meta‐analysis, where the possibility of inconsistency is to be entertained, but it does not provide a very convenient framework for identifying where the inconsistency might arise.

## 6 Simulation study

The proposed method uses the estimated variance components as if they were the true values when making inferences about the treatment effect parameters. This raises the obvious concern that the uncertainty in the parameter estimation is not fully being taken into account. This means that the actual coverage probabilities of confidence intervals are likely to be less than their nominal values, so that the actual significance levels of hypothesis tests are likely to be larger than their nominal values. The aim of the simulation study is to explore this issue. We do not compare the proposed method to an alternative ‘gold standard’ method because there is no currently universally accepted gold standard to compare our procedure to.

### 6.1 The simulation study design

The simulation study imitates previous simulation studies for multivariate meta‐analysis in some respects 9, 10, 36, but we modified previous simulation study designs to make it more suitable for network meta‐analysis. For each study of each design, we simulated a single within‐study variance $\sigma_{\mathrm{di}}^{2}$ from the scaled and truncated *χ* ^2^ distribution originally proposed by Brockell and Gordon 37 for simulating within‐study variances for log odds ratios. Specifically, we simulate within‐study variances as independent $0.25\times \chi_{1}^{2}$ random variables, where we further truncate so that these variances lie between 0.009 and 0.6. The within‐study covariance matrices were then obtained as $S_{\mathrm{di}}=\sigma_{\mathrm{di}}^{2}P_{c_{d}}$. This assumes that, within studies, all within‐study variances are the same and that the within‐study correlations are 0.5. This was deemed appropriate because it reflects an equal allocation of participants to each treatment arm within studies.

In each dataset, we simulated the entire sample of estimates directly from model (8) using an appropriate design matrix and fixed values of $\tau_{\beta }^{2}$ and $\tau_{\omega }^{2}$. We assumed that four treatments appear in the network, so that we estimate three basic parameters *δ* ^*AB*^, *δ* ^*AC*^ and *δ* ^*AD*^. We considered values of 0, 0.024 and 0.168 for both $\tau_{\beta }^{2}$ and $\tau_{\omega }^{2}$, because using these three values of $\tau_{\beta }^{2}$ correspond to *I* ^2^=0,0.3,0.75, where this *I* ^2^ compares the RC and CC models 9, 10, 36. Hence, these values of $\tau_{\beta }^{2}$ can be interpreted as no between‐study heterogeneity, mild heterogeneity and more severe heterogeneity respectively. For $\tau_{\omega }^{2}$, these values correspond to the consistency assumption, mild inconsistency and more severe inconsistency, respectively. Jackson *et al.*, 7 argue that the model should only be used when ‘small amounts of inconsistency are present’. Hence, $\tau_{\omega }^{2}=0.168$ represents a situation where the use of network meta‐analysis *per se* is to be discouraged, but we will investigate what happens if the proposed method is used.

The design matrix **X** depends on the designs of the studies included in the network meta‐analysis, and three simulation runs were performed to explore different possibilities for **X**. The first run involved 10 studies and five different designs. For this, we used two studies of each of the five designs *ABC*, *ABD*, *AB*, *AC* and *AD*, so that in total, there were 14 estimated treatment effects. The second run involved 50 studies and five different designs. For this, we used 10 studies of each of these five designs *ABC*, *ABD*, *AB*, *AC* and *AD*, so that in total, there were 70 estimated treatment effects. The third run involved 50 studies and 10 different designs *ABC*, *ABD*, *ACD*, *BDC*, *AB*, *AC*, *AD*, *BC*, *BD* and *CD* (that is, all possible two and three arm designs were included). For this, we used five studies of each of these 10 designs so that again there were 50 studies that provide 70 treatment effects. The important difference between the second and third runs is that the latter involves more designs so that $\tau_{\omega }^{2}$ can be better estimated. We simulated data assuming all treatment effects are zero, so that in model (8), we use ***δ*** = **0**, but this is immaterial because the estimation of the variance components is location‐invariant and the point estimation of the means is just translated when using an alternative ***δ*** ≠ **0**.

### 6.2 The number of simulated datasets

Three‐thousand independent datasets were simulated for each combination of **X**, $\tau_{\beta }^{2}$ and $\tau_{\omega }^{2}$. Biases were estimated as the average difference between parameter estimates, and the true value and coverage probabilities of nominal 95% confidence intervals were estimated as the proportion of the intervals that cover the true value. Three‐thousand datasets were used because a slight decrease from the nominal coverage probability of 0.95 was expected, and $2\sqrt{0.92\times 0.08/3000}\approx 0.01$, which means that estimated coverage probabilities can be taken to be accurate to within around 0.01 of the correct value.

### 6.3 Results using the full model

For the first two runs, the results for *δ* ^*AC*^ and *δ* ^*AD*^ were in agreement (within Monte Carlo error). This is appropriate because the designs of these two networks are symmetrical in treatments *C* and *D*. For the third run, the results for all three basic parameters were in agreement, which is also appropriate. Hence, we present results for *δ* ^*AB*^ and *δ* ^*AC*^ for the first two runs and the results for *δ* ^*AB*^ only for the third run. Because before truncation the estimates of $\tau_{\beta }^{2}$ and $\tau_{\omega }^{2}$ are obtained by matching moments, they are truly unbiased if truncation is not performed (this was confirmed by the simulation study; results not shown). However, truncation invokes a positive bias in the variance components, and the coverage probabilities of nominal 95% confidence intervals deviated from 0.95 as expected.

Hence, in Table 1, we show the empirical and the average model‐based standard errors of the estimated basic parameters, and the estimated actual coverage probabilities of the corresponding nominal 95% confidence intervals. The model‐based standard errors are obtained from (13) whereas the empirical standard errors are obtained as the standard deviation of the point estimates; if the modelling and estimation are both adequate, then empirical and model‐based standard errors will be in good agreement. We also show the excess kurtosis of the estimates of treatment effect to assess whether the distribution of the estimates have heavier tails than a normal distribution, which would imply that the use of quantiles from a *t* distribution could be more appropriate than those from a standard normal when calculating confidence intervals and performing hypothesis tests. The excess kurtosis is the kurtosis minus 3 (3 is the kurtosis of a normal distribution), so that the excess kurtosis of a normal distribution is 0. Positive values of excess kurtosis suggest that a *t* distribution could perform better than a standard normal distribution when making inferences. Finally, in Table 1, we also show the average (with standard deviations) of the *truncated* estimates of the variance components.

**Table 1:** Results from the simulation study.

Run	$\tau_{\beta }^{2}$	$\tau_{\omega }^{2}$	${\hat{\delta }}^{\mathrm{AB}}$				${\hat{\delta }}^{\mathrm{AC}}$				${\hat{\tau }}_{\beta }^{2}$ (truncated)		${\hat{\tau }}_{\omega }^{2}$ (truncated)	
			SE (emp)	SE (model)	Cover	Excess kurt	SE (emp)	SE (model)	Cover	Excess kurt	E[ ${\hat{\tau }}_{\beta }^{2}$]	SE( ${\hat{\tau }}_{\beta }^{2}$)	E[ ${\hat{\tau }}_{\omega }^{2}$]	SE( ${\hat{\tau }}_{\omega }^{2}$)
1	0	0	0.116	0.149	0.986	0.670	0.149	0.186	0.983	1.003	0.025	0.047	0.021	0.036
1	0.024	0	0.135	0.166	0.971	0.277	0.170	0.204	0.973	0.442	0.043	0.066	0.027	0.045
1	0.168	0	0.216	0.241	0.950	0.095	0.264	0.296	0.945	0.154	0.176	0.168	0.057	0.101
1	0	0.024	0.145	0.166	0.957	0.414	0.180	0.204	0.954	0.217	0.025	0.048	0.038	0.049
1	0.024	0.024	0.165	0.180	0.948	0.173	0.205	0.222	0.942	0.395	0.041	0.062	0.045	0.062
1	0.168	0.024	0.237	0.251	0.937	−0.064	0.282	0.307	0.944	0.010	0.170	0.157	0.076	0.119
1	0	0.168	0.263	0.256	0.911	0.081	0.321	0.314	0.898	0.084	0.026	0.050	0.174	0.166
1	0.024	0.168	0.265	0.261	0.898	−0.015	0.328	0.320	0.898	−0.035	0.041	0.065	0.175	0.172
1	0.168	0.168	0.311	0.308	0.905	0.039	0.377	0.377	0.916	0.051	0.175	0.161	0.194	0.246
2	0	0	0.044	0.051	0.970	0.248	0.054	0.063	0.971	0.136	0.005	0.008	0.002	0.004
2	0.024	0	0.057	0.064	0.958	0.023	0.071	0.078	0.954	0.029	0.024	0.016	0.004	0.006
2	0.168	0	0.096	0.109	0.961	−0.141	0.116	0.133	0.955	−0.057	0.169	0.053	0.013	0.025
2	0	0.024	0.096	0.093	0.900	−0.049	0.118	0.113	0.894	−0.123	0.005	0.008	0.024	0.022
2	0.024	0.024	0.104	0.098	0.888	0.005	0.126	0.120	0.883	−0.011	0.024	0.016	0.025	0.025
2	0.168	0.024	0.128	0.128	0.922	−0.135	0.158	0.157	0.906	−0.118	0.169	0.055	0.031	0.042
2	0	0.168	0.231	0.216	0.874	−0.071	0.278	0.264	0.885	0.101	0.005	0.008	0.168	0.131
2	0.024	0.168	0.232	0.218	0.874	−0.045	0.288	0.267	0.869	−0.020	0.024	0.016	0.168	0.132
2	0.168	0.168	0.245	0.227	0.862	0.070	0.294	0.278	0.865	−0.193	0.168	0.054	0.168	0.153
3	0	0	0.052	0.061	0.975	0.252	—	—	—	—	0.006	0.009	0.003	0.005
3	0.024	0	0.066	0.074	0.964	0.017	—	—	—	—	0.025	0.019	0.005	0.008
3	0.168	0	0.110	0.124	0.960	0.172	—	—	—	—	0.168	0.056	0.016	0.025
3	0	0.024	0.091	0.091	0.932	0.088	—	—	—	—	0.006	0.009	0.024	0.017
3	0.024	0.024	0.100	0.098	0.932	−0.003	—	—	—	—	0.025	0.019	0.025	0.021
3	0.168	0.024	0.133	0.137	0.940	0.027	—	—	—	—	0.168	0.056	0.033	0.043
3	0	0.168	0.198	0.194	0.920	−0.020	—	—	—	—	0.006	0.009	0.166	0.082
3	0.024	0.168	0.200	0.198	0.916	−0.054	—	—	—	—	0.025	0.018	0.169	0.088
3	0.168	0.168	0.222	0.215	0.911	−0.013	—	—	—	—	0.167	0.057	0.170	0.114

Run 1 has 10 studies of five different designs, run 2 has 50 studies of five different designs and run 3 has 50 studies of 10 different designs. SE (emp) is the empirical standard error of the estimates, and SE (model) is the average model‐based standard error. Cover is the estimated coverage probability of nominal 95% confidence intervals, and Excess kurt is the excess kurtosis. The average truncated ${\hat{\tau }}_{\beta }^{2}$ and ${\hat{\tau }}_{\omega }^{2}$, and the empirical standard errors of these estimates, are also shown as E and SE, respectively. For each of the 27 sets of parameter values used, 3000 simulated datasets were produced. In runs 1 and 2, the results for ${\hat{\delta }}^{\mathrm{AD}}$ are obtained as those of ${\hat{\delta }}^{\mathrm{AC}}$ due to the symmetries of the networks in treatments *C* and *D*. Similarly, in run 3, the results for both ${\hat{\delta }}^{\mathrm{AC}}$ and ${\hat{\delta }}^{\mathrm{AD}}$ are obtained as those of ${\hat{\delta }}^{\mathrm{AB}}$.

From Table 1, we see that the truncation of the variance components results in notable positive bias when the sample size is small (run 1), but this bias is much smaller in runs 2 and 3. The proposed approach is conservative (the actual coverage probabilities of the confidence intervals are larger than the nominal 0.95) when the consistency assumption is true $\left(\tau_{\omega }^{2}=0\right)$, as one should anticipate, because then the model allows for a superfluous variance component. This is reminiscent of the conservatism of the univariate random effects model when the fixed effect assumption is true. When the inconsistency is present ( $\tau_{\omega }^{2}=0.024,0.168$), the actual coverage probabilities drop from the nominal 0.95. This is because the proposed method does not take into account the uncertainty in the estimated variance components. Given the simplicity of the method, the general picture from Table 1 is that the method performs reasonably well. The proposed method is conservative when the empirical standard errors are less than the average model‐based standard errors and is anti‐conservative when the empirical standard errors are more than the average model‐based standard errors, as expected.

It is interesting, and perhaps slightly disconcerting, that actual coverage probabilities in run 2 are not better than those from run 1 and, in fact, appear to be slightly worse. This is despite the fact that run 2 involves 50 studies but run 1 only involves 10 studies. This can be explained because run 2 involves the same number of designs as run 1, so the estimation of $\tau_{\omega }^{2}$ is not impressively better in run 2 than in run 1; this can be seen from the magnitudes of the standard deviations of the estimates of the inconsistency variances. The precision of the estimated treatment effects becomes considerably greater in run 2 as expected, but the magnitude of the uncertainty in $\tau_{\omega }^{2}$ relative to this precision remains large in run 2. Hence, the impact of taking $\tau_{\omega }^{2}$ as fixed and known remains considerable in run 2. This is because in moving from run 1 to run 2, we have begun to take a limit in an artificial way: we have begun to allow the number of studies to tend towards infinity while holding the number of designs fixed. For the proposed approach to perform well, we require that both variance components are well estimated, so that ideally, we need large numbers of studies *and* designs. This is the case in run 3, where we have 50 studies and 10 designs. The actual coverage probabilities in run 3 are closer to the nominal level than in runs 1 and 2, which emphasises that the amount of replication within and across designs together determine the accuracy of the proposed method in practice.

The values of excess kurtosis in Table 1 are both positive and negative, which makes it unlikely that a consistent improvement could be obtained simply by using quantiles from a *t* distribution, rather than a standard normal, when making inferences. However, the excess kurtosis is generally greatest when both $\tau_{\beta }^{2}$ and $\tau_{\omega }^{2}$ are zero, which suggests that the distributions of the estimates of treatment become heavy‐tailed when superfluous variance components are included in the model.

### 6.4 The implications of making the consistency assumption

The consistency assumption is commonly, and sometimes implicitly, made. In Table 2, we present the results where we apply the consistency analysis described in Section 4.1 to the simulated datasets (we omit the results $\tau_{\omega }^{2}$ because this is assumed to be zero in a consistency analysis). Hence, we calculate ${\hat{\tau }}_{\beta }^{2}$ from Equation (10) with ${\hat{\tau }}_{\omega }^{2}$ constrained to be zero.

**Table 2:** Results from the simulation study where the consistency model is fitted.

Run	$\tau_{\beta }^{2}$	$\tau_{\omega }^{2}$	${\hat{\delta }}^{\mathrm{AB}}$				${\hat{\delta }}^{\mathrm{AC}}$				${\hat{\tau }}_{\beta }^{2}$	
			SE (emp)	SE (model)	Cover	Excess kurt	SE (emp)	SE (model)	Cover	Excess kurt	E[ ${\hat{\tau }}_{\beta }^{2}$]	SE( ${\hat{\tau }}_{\beta }^{2}$)
1	0	0	0.110	0.116	0.965	0.775	0.144	0.148	0.959	1.169	0.015	0.029
1	0.024	0	0.133	0.132	0.921	0.263	0.167	0.164	0.924	0.427	0.033	0.042
1	0.168	0	0.215	0.202	0.910	0.091	0.262	0.248	0.913	0.139	0.167	0.125
1	0	0.024	0.143	0.127	0.898	0.390	0.178	0.158	0.902	0.263	0.027	0.039
1	0.024	0.024	0.164	0.142	0.889	0.161	0.204	0.177	0.884	0.370	0.048	0.052
1	0.168	0.024	0.236	0.209	0.890	−0.037	0.281	0.256	0.905	0.024	0.184	0.132
1	0	0.168	0.267	0.183	0.791	0.045	0.325	0.225	0.795	0.074	0.129	0.124
1	0.024	0.168	0.269	0.192	0.804	−0.039	0.329	0.237	0.803	−0.056	0.149	0.134
1	0.168	0.168	0.313	0.245	0.842	0.050	0.378	0.299	0.851	0.038	0.290	0.212
2	0	0	0.044	0.045	0.953	0.235	0.054	0.055	0.950	0.152	0.004	0.007
2	0.024	0	0.057	0.055	0.934	0.014	0.071	0.068	0.932	0.017	0.024	0.015
2	0.168	0	0.096	0.093	0.941	−0.145	0.116	0.114	0.942	−0.048	0.169	0.052
2	0	0.024	0.097	0.051	0.688	−0.036	0.119	0.063	0.690	−0.091	0.016	0.016
2	0.024	0.024	0.105	0.061	0.739	0.017	0.127	0.075	0.752	−0.006	0.038	0.022
2	0.168	0.024	0.128	0.096	0.850	−0.143	0.158	0.117	0.843	−0.125	0.183	0.058
2	0	0.168	0.233	0.077	0.468	−0.114	0.281	0.094	0.484	0.116	0.101	0.079
2	0.024	0.168	0.234	0.083	0.503	−0.033	0.289	0.101	0.506	−0.037	0.124	0.080
2	0.168	0.168	0.246	0.109	0.605	0.054	0.294	0.133	0.606	−0.197	0.268	0.105
3	0	0	0.051	0.054	0.961	0.284	—	—	—	—	0.004	0.007
3	0.024	0	0.066	0.066	0.944	−0.044	—	—	—	—	0.025	0.016
3	0.168	0	0.110	0.110	0.939	0.167	—	—	—	—	0.168	0.051
3	0	0.024	0.092	0.064	0.815	0.067	—	—	—	—	0.020	0.016
3	0.024	0.024	0.101	0.075	0.848	0.030	—	—	—	—	0.044	0.021
3	0.168	0.024	0.133	0.115	0.901	0.024	—	—	—	—	0.188	0.058
3	0	0.168	0.203	0.102	0.674	−0.067	—	—	—	—	0.137	0.069
3	0.024	0.168	0.203	0.108	0.694	−0.110	—	—	—	—	0.163	0.073
3	0.168	0.168	0.223	0.136	0.757	−0.048	—	—	—	—	0.306	0.101

Run 1 has 10 studies of five different designs, run 2 has 50 studies of five different designs and run 3 has 50 studies of 10 different designs. SE (emp) is the empirical standard error of the estimates, and SE (model) is the average model‐based standard error. Cover is the estimated coverage probability of nominal 95% confidence intervals, and Excess kurt is the excess kurtosis. The average truncated ${\hat{\tau }}_{\beta }^{2}$, and the empirical standard error of these estimates, are also shown as E and SE, respectively. For each of the 27 sets of parameter values used, 3000 simulated datasets were produced. In runs 1 and 2, the results for ${\hat{\delta }}^{\mathrm{AD}}$ are obtained as those of ${\hat{\delta }}^{\mathrm{AC}}$ due to the symmetries of the networks in treatments *C* and *D*. Similarly, in run 3, the results for both ${\hat{\delta }}^{\mathrm{AC}}$ and ${\hat{\delta }}^{\mathrm{AD}}$ are obtained as those of ${\hat{\delta }}^{\mathrm{AB}}$.

From Table 2, we can see that the consistency analysis is less conservative than the proposed method (Table 1) when the consistency assumption is true ( $\tau_{\omega }^{2}=0$). The consistency analysis also results in more precise estimation of $\tau_{\beta }^{2}$ when the consistency assumption is true. However, the consistency analysis becomes misleading when inconsistency is present. When $\tau_{\omega }^{2}>0$, the truncated ${\hat{\tau }}_{\beta }^{2}$ becomes even more positively biased, because the consistency model attempts to describe the additional variation in the data. Despite this, the consistency model fails to adequately describe the data and the nominal coverage probability drops accordingly. The results in Table 2 make it clear that analyses that incorrectly make the consistency assumption can be misleading.

To summarise, the simulation study reassures us that the proposed method performs adequately. Indeed, the method performs better than many might suspect given its simplicity. However, it requires sufficient replication both within and between designs to perform very well. Because the proposed method is conservative when the consistency assumption is true, it provides a very viable option under the assumption of consistency or when a small amount of inconsistency is present. These are exactly the circumstances under which the use of network meta‐analysis should be encouraged, and so the proposed method performs best in the situations where we anticipate that most network meta‐analyses will be performed.

## 7 Examples

In this section, we apply our new method to two real examples and we compare the results to those obtained using Bayesian analyses performed using WinBUGS. Details of the prior specification used for the first example (Section 7.1 in the succeeding text) are given by Jackson *et al*. 7, and the same priors were used for the second example (Section 7.2), but we also briefly give details here. All location parameters were given vague (normal with very small precision) prior distributions. Uniform prior distributions from 0 to 5 were used for the standard deviations *τ*_*β*_ and *τ*_*ω*_. Because the prior distributions used for variance components are often influential in Bayesian analyses, these particular priors are only intended to provide some illustrative results. Investigating the sensitivity of conclusions to the prior distributions is highly recommended in practice.

A very large burn‐in of 30 000 was used for the first example to ensure convergence, which was checked by running three chains at different starting values. The second example is of much lower dimension and presents considerably less of a challenge for Markov chain monte carlo (MCMC) algorithms, so here, Bayesian analyses were performed using single chains with burn‐ins of 20 000. For making inferences for the first and second examples, 450 000 and 100 000 draws from the posterior distributions were used, respectively.

### 7.1 Example one: osteoarthritis of the knee

This example was used previously by Jackson *et al*. 7. Here, there are 87 trials of 38 designs, each comparing a subset of 22 treatments for pain relief for osteoarthritis of the knee. The outcomes are continuous measurements, and the standardised mean difference of pain at the end of the trial is used to compare treatments, where a negative treatment effect indicates benefit. For one trial, only treatment contrasts were available, and two arms were in the same treatment category. Jackson *et al.*, 7 treated these treatment effects as from a multi‐arm trial with a shared random effect contributing to each treatment effect. The proposed approach does not allow this type of flexibility in the modelling, and here, we instead chose one of the treatment effects to use in the analysis at random. This example involves many different studies and designs, but there is relatively little replication of studies within designs.

The results are shown in Table 3. The previous WinBUGS analysis reported the results in terms of the standard deviations *τ*_*β*_ and *τ*_*ω*_ so in Table 3, we present the truncated estimates ${\hat{\tau }}_{\beta }$ and ${\hat{\tau }}_{\omega }$, which were obtained by square‐rooting the moments‐based estimates of the two variance parameters. The probabilities that each treatment is best were obtained as explained in Section 5.2 using 10 000 simulated treatment effect vectors. The two sets of results are in reasonable agreement, but the main difference is that the proposed method provides a truncated estimate ${\hat{\tau }}_{\omega }^{2}=0$. This is not surprising because the DIC statistic from the Bayesian analysis of these data assuming consistency is smaller than the corresponding DIC for the full model (132.61 vs. 133.23) 7. This indicates that the consistency model adequately describes the data as reflected in the truncated ${\hat{\tau }}_{\omega }^{2}=0$. However, this results in smaller standard errors for the estimated basic parameters, and the inferences from the proposed method are in better agreement with the previous Bayesian analysis where consistency was assumed 7.

**Table 3:** Results for the osteoarthritis of the knee data.

Treatment	Parameter	New procedure		Previous Bayesian analysis	
		Estimate	*P* (best)	Estimate	*P* (best)
A: standard care	—		0.00		0.00
B: placebo	*δ* ^*AB*^	0.04 (0.20)	0.00	0.04 (0.23)	0.00
C: no medication	*δ* ^*AC*^	0.60 (0.32)	0.00	0.59 (0.35)	0.00
D: acupuncture	*δ* ^*AD*^	−0.78 (0.16)	0.08	−0.78 (0.19)	0.07
E: balneotherapy	*δ* ^*AE*^	−0.46 (0.25)	0.00	−0.49 (0.30)	0.01
F: braces	*δ* ^*AF*^	−0.15 (0.46)	0.02	−0.15 (0.50)	0.02
G: aerobic exercise	*δ* ^*AG*^	−0.59 (0.22)	0.03	−0.57 (0.25)	0.03
H: muscle exercise	*δ* ^*AH*^	−0.37 (0.11)	0.00	−0.36 (0.15)	0.00
I: heat treatment	*δ* ^*AI*^	−0.03 (0.30)	0.00	−0.02 (0.33)	0.00
J: insoles	*δ* ^*AJ*^	−0.01 (0.35)	0.00	0.00 (0.41)	0.00
K: tai chi	*δ* ^*AK*^	−0.28 (0.29)	0.01	−0.28 (0.34)	0.01
L: weight loss	*δ* ^*AL*^	−0.35 (0.26)	0.01	−0.35 (0.29)	0.01
M: sham acupuncture	*δ* ^*AM*^	−0.25 (0.23)	0.00	−0.28 (0.28)	0.00
N: ice/cooling	*δ* ^*AN*^	−0.25 (0.37)	0.01	−0.25 (0.39)	0.01
O: interferential	*δ* ^*AO*^	−1.11 (0.48)	0.52	−1.11 (0.51)	0.49
P: laser	*δ* ^*AP*^	−0.25 (0.36)	0.00	−0.24 (0.42)	0.01
Q: manual	*δ* ^*AQ*^	−0.29 (0.30)	0.00	−0.29 (0.32)	0.01
R: NMES	*δ* ^*AR*^	0.46 (0.55)	0.00	0.45 (0.58)	0.00
S: PES	*δ* ^*AS*^	−0.70 (0.32)	0.08	−0.73 (0.36)	0.09
T: PEMF	*δ* ^*AT*^	0.01 (0.32)	0.00	0.01 (0.38)	0.00
U: static magnets	*δ* ^*AU*^	−0.78 (0.57)	0.24	−0.78 (0.61)	0.23
V: TENS	*δ* ^*AV*^	−0.63 (0.22)	0.01	−0.61 (0.24)	0.01
Heterogeneity	*τ*_*β*_	0.42		0.42 (0.06)	
Inconsistency	*τ*_*ω*_	0		0.14 (0.10)	

NMES, neuromuscular electrical stimulation; PES, pulsed electrical stimulation; PEMF, pulsed electro‐ magnetic fields; TENS, transcutaneous electrical nerve stimulation.Two sets of results are shown, those using the new ‘DerSimonian and Laird’ method and those from the previous analysis using WinBUGS are also shown for comparison. For the new procedure, estimates are followed by standard errors in parentheses. For the previous WinBUGS analysis, the estimates are posterior means, which are followed by posterior standard deviations in parentheses. *P* (best) is the probability that each treatment is best; these probabilities are included so that the results using the new method can be compared with those obtained previously and are not adequate for the full probabilistic ranking of the treatments.

The results for this example in Table 3 are therefore in reasonable agreement with the results obtained previously, and the discrepancies are easily explained. However, the proposed method appears to understate the uncertainty in the results from an analysis that allows for the possibility of inconsistency. This is despite the relatively large sample size of the osteoarthritis of the knee dataset. The *I* ^2^ statistics however are quite similar to those obtained previously using WinBUGS: the *I* ^2^ statistic comparing the *RI* and *RC* models is 12%, the *I* ^2^ statistic comparing the *RI* and the *CC* models is 71% and the *I* ^2^ statistic comparing the *RC* and the *CC* models is 68%; in the previous WinBUGS analyses, these three *I* ^2^ statistics were 19%, 75% and 69%, respectively.

### 7.2 Example two: topical antibiotics

This example is taken from a Cochrane Systematic Review *Topical antibiotics without steroids for chronically discharging ears with underlying eardrum perforations* 38. Here, 13 studies, two of which are three‐arm studies, provide outcome data on treatment failure due to persistent discharge. The network meta‐analysis involves four treatment arms: A (no treatment), B (topical quinolone antibiotic), C (topical non‐quinolone antibiotic) and D (topical antiseptic). Unlike the previous example, here, binary outcome data are available. Hence, when using the Bayesian implementation of the model proposed by Jackson *et al*. 7, exact binomial within‐study distributions can be used in an arm‐based analysis as explained in section 2.6 of their paper. Hence, when using WinBUGS to produce results to compare those from the new method with, both these binomial distributions and normal approximations to the log odds ratios were used. The Bayesian results using binomial distributions give us an indication of the adequacy of the normal approximations used in the other two analyses. The variances of the log odds ratios used in the normal approximations were obtained in the usual way, and the within‐study covariances were obtained as the variance of the log odds in the baseline treatment arm. The proposed method uses the normal approximations as explained in Section 2.4. Because the treatment effects are log odds ratios comparing the other treatments to treatment *A*, and the outcome is harmful, negative basic parameters indicate that treatments *B*, *C* and *D* are more beneficial than treatment *A* (no treatment).

The results are shown in Table 4, which sets out the results in the same way as in Table 3. Comparing the two sets of WinBUGS results, we can see that the use of normal approximations dilutes the estimated treatment effects, which shows that the use of normal approximations can have quite a considerable impact unless the studies are very large. The proposed procedure further dilutes the estimated treatment effects, and the standard errors are very considerably smaller than the posterior standard deviations from the Bayesian analyses. One reason why the proposed method results in smaller standard errors is because it does not take into account the uncertainty in the variance components, which is very considerable in relatively small and highly inconsistent networks such as this one. However, the proposed method also results in a much smaller ${\hat{\tau }}_{\omega }$ than the Bayesian analyses, and this too reduces the standard errors. Better agreement can be expected in situations where the estimated variance components using the various estimation methods are smaller and more similar. Although the simulation study reassures us that the repeated sampling properties of the proposed method are not unacceptable in small networks, this example provides a pertinent reminder that the proposed method will, in general, result in artificially small standard errors of the treatment effects unless the network is large. Methods that attempt to take into account the uncertainty in variance components, which are analogous to those proposed in multivariate meta‐analysis 36, 39, are clearly worth developing for use in examples such as this one but are beyond the scope of this paper.

**Table 4:** Results for the topical antibiotics data.

Treatment	Parameter	New procedure Estimate	Bayesian (normal) Estimate	Bayesian (binomial) Estimate
B: quinolone	*δ* ^*AB*^	−1.92 (0.64)	−2.07 (1.09)	−2.26 (1.11)
C: non‐quinolone	*δ* ^*AC*^	−1.35 (0.74)	−1.46 (1.21)	−1.65 (1.30)
D: antiseptic	*δ* ^*AD*^	−0.67 (0.67)	−0.77 (1.15)	−0.93 (1.13)
Heterogeneity	*τ*_*β*_	0.50	0.56 (0.37)	0.68 (0.38)
Inconsistency	*τ*_*ω*_	0.55	0.96 (0.70)	1.11 (0.74)

Three sets of results are shown, those using the new method and then two sets of results using WinBUGS are also shown for comparison: those using normal approximations (normal) and those using binomial within‐study distributions. For the new ‘DerSimonian and Laird’ procedure, estimates are followed by standard errors in parentheses. For the WinBUGS analyses, the estimates are posterior means, which are followed by posterior standard deviations in parentheses.

The *I* ^2^ statistics are generally reasonably similar to those obtained from the Bayesian analyses. Using the proposed method, the *I* ^2^ statistic comparing the *RI* and *RC* models is 30%, the *I* ^2^ statistic comparing the *RI* and the *CC* models is 80% and the *I* ^2^ statistic comparing the *RC* and the *CC* models is 72%. In the Bayesian analysis using normal approximations, these *I* ^2^ statistics are 56%, 92% and 81%, respectively. In the Bayesian analysis using binomial within‐study distributions, these *I* ^2^ statistics are 66%, 93% and 80%, respectively.

## 8 Discussion

We have proposed an extension of DerSimonian and Laird's procedure for random effects meta‐analysis that can be used for network meta‐analysis. Our method retains all the advantages of DerSimonian and Laird's approach but also inherits all its limitations. Furthermore, by extending DerSimonian and Laird's method to a model for network meta‐analysis with two random effects, the limitations of this method might be thought to be exacerbated, but the simulation study reassures us that the proposed method performs adequately. The main limitations are that normal approximations are used, reasonably large datasets are required for accurate inference and the estimation of the variance components is not based on sufficient statistics, so that more precise inferences for $\tau_{\beta }^{2}$ and $\tau_{\omega }^{2}$ are likely to be possible using likelihood‐based methods. The question of how to make good inference about the uncertainty in the estimated variance components remains an open one, but some form of bootstrapping provides a practical approach. The second example illustrates that the use of normal approximations can have considerable impact, but our simulation study reassures us that the repeated sampling properties of the proposed method are reasonable when these normal approximations are adequate. The simulation study suggests that five different designs is probably the minimum number required to adequately estimate the inconsistency variance. Sufficient replication is also required within designs in order to adequately estimate the between‐study heterogeneity variance. The proposed method only works very well when the departure from the consistency assumption is small, but network meta‐analyses are, in any case, to be generally discouraged when the inconsistency in the network is large. The main advantages of the proposed method is that no iteration is required, the estimation is simple and transparent and that it is closely related to methods that many working in meta‐analysis will already be familiar with. Another advantage is that the method is semi‐parametric (the formula for the expectation of a quadratic form used in the Appendices does not require the normality assumption (Searle 40, p. 55)) so that valid meta‐analyses can be performed in large samples without requiring the assumption that either the between‐study heterogeneity or the inconsistency follow normal distributions.

Our estimation method is based on the recently proposed decomposition of the *Q* statistic. Alternative quadratic forms could also be used and warrant further investigation. More general variance structures for the between‐study heterogeneity and inconsistency parameters should be considered in datasets where these may be well identified, and extending the method to accommodate them also warrants further investigation. Likelihood‐based frequentist methods could easily accommodate alternative assumptions for the random effects but have the disadvantage of requiring iterative methods to maximise the likelihood. Although the proposed model is a type of linear mixed model, models for meta‐analysis such as ours require the use of fixed and known within‐study variances. This makes many standard algorithms for linear mixed models either inappropriate or difficult to use. The developing of likelihood‐based methods for fitting the model, and variants of this model, is left as an important avenue for further work.

Most of the types of inference that are used in the context of likelihood‐based or Bayesian analyses are also available when using the method of moments in the way we propose, but no DIC or AIC model fit statistics are immediate. This is one of the prices we pay for using the method of moments rather than a likelihood‐based or a Bayesian analysis. Here, we focus on a new method for performing the estimation, but appropriate graphical displays 41 are important in practice to help visualise the data and informally assess the model fit. Determining the most appropriate graphical displays to accompany our method may form the subject of future work.

We have used random effects for the inconsistency parameters, but fixed effects, or node splitting, are probably more suitable for exploring where any inconsistency in the network arises. White *et al.*, 23 describe how fixed effects inconsistency parameters can be used in the context of multivariate meta‐regression models, and we refer the reader to this approach if a frequentist network meta‐analysis, similar to ours but with fixed effects inconsistency parameters, is to be used.

To summarise, we have proposed a simple, transparent and direct method for network meta‐analysis that we believe is accessible to applied researchers. An *R* function is available in the Supporting Information that implements the proposed method, and we hope that this will also serve to make our method attractive to the meta‐analysis community.

## Supporting information



Supporting info item

## Appendix A

### A.1

We define ${\hat{\beta }}_{d}$ as the estimate under model (3) where $\tau_{\beta }^{2}=0$. From standard regression models with correlated errors, we have that ${\hat{\beta }}_{d}={\left(X_{d}^{T}W_{d}X_{d}\right)}^{-1}X_{d}^{T}W_{d}Y_{d}$ where $W_{\mathrm{di}}=S_{\mathrm{di}}^{-1}$ and ***W***_*d*_=diag(***W***_*d**i*_); that is, ***W***_*d*_ is the block diagonal matrix containing the ***W***_*d**i*_. In order to derive the properties of (4), we write this in matrix form. We have that

$$Y_{d}-{\hat{Y}}_{d}=\left(I_{n_{d}c_{d}}-X_{d}{\left(X_{d}^{T}W_{d}X_{d}\right)}^{-1}X_{d}^{T}W_{d}\right)Y_{d}$$

and (4) can be written as

$$Q_{d}^{\text{het}}={\left(Y_{d}-{\hat{Y}}_{d}\right)}^{T}W_{d}(Y_{d}-{\hat{Y}}_{d}).$$

After a little manipulation, we can write

(A.1) $$Q_{d}^{\text{het}}=Y_{d}^{T}B_{d}Y_{d}$$

where $B_{d}=W_{d}-W_{d}X_{d}{\left(X_{d}^{T}W_{d}X_{d}\right)}^{-1}X_{d}^{T}W_{d}$. Substituting **Y**_*d*_=**X**_*d*_ ***β***_*d*_+**Z**_*d*_, where $Z_{d}∼N\left(0,S_{d}+\tau_{\beta }^{2}{\text{I}}_{n_{d}}⊗{\text{P}}_{c_{d}}\right)$, into (A.1), and using the properties of ***B***_*d*_ give

(A.2) $$Q_{d}^{\text{het}}=Z_{d}^{T}B_{d}Z_{d}$$

Searle 40, theorem 1 on p. 55, implies that when **x** ∼ *N*(**0**,**V**), then E[**x** ^*T*^ **A** **x**] = tr(**A** **V**). Then applying this result to (A.2) means that

(A.3) $$E\left[Q_{d}^{\text{het}}\right]=\text{tr}\left(B_{d}\left({\text{S}}_{d}+\tau_{\beta }^{2}{\text{I}}_{n_{d}}⊗{\text{P}}_{c_{d}}\right)\right)$$

Then we have $\text{tr}(B_{d}S_{d})=\text{tr}(I_{n_{d}c_{d}}-H_{d})$ where **H**_*d*_ is the hat matrix corresponding to a standard unweighted regression where the design matrix is $W_{d}^{1/2}X_{d}$. This means that tr(**B**_*d*_ **S**_*d*_) = (*n*_*d*_−1)*c*_*d*_, which is the degrees of freedom associated with the regression. This result and (A.3) immediately result in (5).

## Appendix B

### B.1

This appendix proceeds in a very similar way to the previous one. We define $\hat{\delta }$ as the estimate under model (8) where $\tau_{\beta }^{2}=\tau_{\omega }^{2}=0$. From standard regression models with correlated errors, we have that $\hat{\delta }={\left(X^{T}WX\right)}^{-1}X^{T}WY$ where ***W*** = diag(***W***_*d*_). In order to derive the properties of *Q* ^net^, we write this in matrix form. We have that

$$Y-\hat{Y}=(I_{a}-X{\left(X^{T}WX\right)}^{-1}X^{T}W)Y,$$

where *a* is the dimension of ***Y*** so that *a* is the total number of estimates from all studies of all designs, and *Q* ^net^ can be written as

$$Q^{\text{net}}={\left(Y-\hat{Y}\right)}^{T}W(Y-\hat{Y})$$

After a little manipulation, we can write

(B.1) $$Q^{\text{net}}=Y^{T}BY$$

where ***B*** = ***W*** − ***W*** ***X***(***X*** ^*T*^ ***W*** ***X***)^−1^ ***X*** ^*T*^ ***W***. Substituting **Y** = **X** ***δ*** + **Z**, where $Z∼N\left(0,S+\tau_{\beta }^{2}{\text{P}}_{1}+\tau_{\omega }^{2}{\text{P}}_{2}\right)$, into (B.1), and using the properties of ***B*** give

$$Q^{\text{net}}=Z^{T}BZ$$

Then applying Searle 40, theorem 1 on p. 55, implies that

$$E[Q^{\text{net}}]=\text{tr}(B(S+\tau_{\beta }^{2}{\text{P}}_{1}+\tau_{\omega }^{2}{\text{P}}_{2}))$$

As in the previous Appendix, we obtain tr(***B***(**S**)) as the degrees of freedom in the regression so that

$$E[Q^{\text{net}}]=\left(\overset{D}{\underset{d=1}{\sum }}n_{d}c_{d}\right)-c+\tau_{\beta }^{2}\text{tr}({\text{BP}}_{1})+\tau_{\omega }^{2}\text{tr}({\text{BP}}_{2})$$

as given in Equation (9).

## Appendix C

### C.1

Recalling that ***X***_*d*_ is obtained by stacking identity matrices, we can continue from Appendix A and evaluate ***B***_*d*_=***W***_*d*_−***M***_*d*_, where the *i*th by *j*th block of ***M***_*d*_ is equal to $W_{\mathrm{di}}W_{d+}^{-1}W_{\mathrm{dj}}$, where $W_{d+}^{-1}$ denotes the sum of the $W_{\mathrm{di}}^{-1}$ matrices for studies of design *d*. Then (A.3), after some algebra that makes use of $S_{d}^{-1}=W_{d}$ and the definition ${\text{P}}_{c_{d}}=({\text{I}}_{c_{d}}+{\text{J}}_{c_{d}})/2$, results in an equivalent formula for *K*_*d*_ given by

$$K_{d}=\frac{1}{2}\left(\text{tr}\left(\overset{n_{d}}{\underset{i=1}{\sum }}W_{\mathrm{di}}+\overset{n_{d}}{\underset{i=1}{\sum }}W_{\mathrm{di}}J_{c_{d}}\right)-\text{tr}\left(\overset{n_{d}}{\underset{i=1}{\sum }}W_{\mathrm{di}}W_{d+}^{-1}W_{\mathrm{di}}+\overset{n_{d}}{\underset{i=1}{\sum }}W_{\mathrm{di}}W_{d+}^{-1}W_{\mathrm{di}}J_{c_{d}}\right)\right)$$

This is a much more obvious generalisation of the coefficient in the standard estimating equation provided by DerSimonian and Laird 8. If all studies are two‐arm trials of the same design, we have $Q^{\text{net}}=\sum \overset{\text{het}}{\underset{d}{Q}}=Q$, where *Q* is Cochran's *Q* statistic and *Q* ^inc^=0. Furthermore, all the **W**_*d**i*_=*w*_*i*_ are scalars, *c*_1_=1, and Equation (5) becomes the familiar

$$E[Q]=(n-1)+\left(\sum w_{i}+\frac{\sum \overset{2}{\underset{i}{w}}}{\sum w_{i}}\right)\tau^{2}$$

where *n* = *n*_1_ and $\tau^{2}=\tau_{\beta }^{2}$. This proves that the proposed method reduces to the method proposed by DerSimonian and Laird and so is an extension of this for network meta‐analysis as stated.
