# Supplementary material for: Extending DerSimonian and Laird's methodology to perform network meta‐analyses with random inconsistency effects
Source: Stat Med. 2015 Sep 30;35(6):819–39. doi: 10.1002/sim.6752 (PMC4973704; doi:10.1002/sim.6752)
Supplement: Supplementary file 1 — Supporting info item [file SIM-35-819-s001.docx]

**Supplementary information: Extending DerSimonian and Laird's methodology to perform network meta-analyses with random inconsistency effects**

**R function for all applications**

The function takes a single argument, a list. This list contains two further lists, y and s, and the vector d, all of length equal to the total number of studies. The list y contains the estimated treatment effects *y_d_*_i_, which are scalars if there is only one effect (e.g. design *BC*), vectors otherwise. The list s contains the within-study covariance matrices *s_di_*, which again may be scalars if there is only one effect. The vector d contains the design of each study, (e.g. *AB*, *AC*, *BDE*). Each treatment should be allocated a letter, with *A* being the reference treatment. The notation closely follows that of the main paper.

The main results included in the output are: est, the estimated treatment effects; Cov, the covariance matrix for the active treatments; se, the standard error of each estimated treatment effect; p, the associated p values; tau2beta, tau2omega, the (*untruncated*) estimates of the variance components $\tau_{\beta}^{2}$ and $\tau_{\omega}^{2}$ respectively. The truncated estimates can of course be obtained from their untruncated counterparts.

Corresponding estimates are also obtained under the assumption of consistency (i.e. $\tau_{\omega}^{2}$ is assumed to equal zero – the "random-effects and consistent" model); these results are appended with “_con”. Finally, the *I^2^* statistics are returned: *I_12_^2^* compares the random-effects and consistent model with the “common-effect and consistent” model; *I_13_^2^* compares the main random-effects and inconsistent model with the common-effect and consistent model, and *I_23_^2^* compares the main random-effects and inconsistent model with the random-effects and consistent model.

*The Matrix package is required for the program to run.*

Cut and paste the code below into R to produce the main results for the OAK data.

fn <- function(all_data){

require(Matrix) # Necessary for bdiag function

n <- length(unlist(all_data$y)) # Total number of treatment effects

smat=bdiag(all_data$s) # Create and fill matrix S

############## Create and fill matrix X ##############

trts <- 0 # Number of treatments, including placebo

for(i in 1:26) trts <- trts + grepl(LETTERS[i], paste(all_data$d,collapse=""))

X=NULL

for(i in 1:length(all_data$d))

{ # For each study d, create a small matrix [no. active trts in study i] x [total no. trts-1].

des_letters=strsplit(all_data$d[i], "")[[1]]

des_numbers=match(des_letters, LETTERS)

cons=length(des_numbers)-1

X1=matrix(0, nrow=cons, ncol=trts) # Create the empty X1 matrix;

for(j in 1:cons) X1[j, des_numbers[j+1]]=1 # Add 1's in the columns of active treatments;

X1[,des_numbers[1]]=-1 # Add a -1 in the column of the (relative) reference trt;

X1 <- X1[, -1]

X=rbind(X, X1, deparse.level = 0) # Add small X1 matrices to create full design matrix X

}

############## Create matrix P1 ##############

p1 <- smat # Same zero terms as S, but

p1[which(p1[,]!=0)] <- 0.5 # all non-zero terms should equal 0.5, except

diag(p1) <- 1 # the diagonal, which should equal 1

############# Create matrix P2 ################

designs=NULL

for(i in 1:length(all_data$y))

{

new_designs=rep(all_data$d[i], length(all_data$y[[i]]))

designs=c(designs, new_designs)

}

p2 <- matrix(0, nrow=n, ncol=n)

for(i in 1:n)

for(j in 1:n)

{

if(designs[i]==designs[j] & sum(X[i,]==X[j,])==trts-1) p2[i,j]=1

if(designs[i]==designs[j] & sum(X[i,]==X[j,])!=trts-1) p2[i,j]=0.5

}

################# Find Qhet_d, where #################

# Qhet_d = Y_d' B_d Y_d

# (see Appendix 1)

#dof = n-trts+1 # Degrees of freedom

unique_d <- unique(all_data$d) # Each unique design

d <- length(unique_d) # Number of unique studies

q_list <- list(qhet=NULL, dof=NULL, k=NULL, ndcd=NULL)

for(i in 1:d){ # For design i, record:

nd <- sum(unique_d[i]==all_data$d) # Number of studies

which_d <- which(unique_d[i]==all_data$d) # Which studies have design i?

yd <- unlist(all_data$y[which_d]) # Treatment effects assoc'd with studies of design i

cd <- nchar(unique_d[i])-1 # No. of treatments compared to reference treatment

icd <- diag(cd) # Identity matrix Icd

xd <- matrix(rep(icd, nd), ncol=cd, byrow=T) # Stacked identity matrices

sd <- smat[which(unique_d[i]==designs), which(unique_d[i]==designs)] # Block diag. matrix containing S_di matrices

wd <- solve(sd)

pcd <- matrix(rep(0.5, cd*cd), nrow=cd)

diag(pcd) <- 1

ind <- diag(nd) # Identity matrix with dim=number of studies

kron <- ind %x% pcd # Kronecker product I_nd (x) P_cd (see equation 17)

bd <- wd - wd %*% xd %*% solve(t(xd) %*% wd %*% xd) %*% t(xd) %*% wd

kd <- sum(diag(bd %*% kron))

q_list$qhet[i] <- as.numeric(t(yd) %*% bd %*% yd)

q_list$dof[i] <- (nd-1)*cd

q_list$k[i] <- kd

q_list$ndcd[i] <- nd*cd

}

################## Find tau2_beta #####################

qhet <- sum(q_list$qhet)

dof <- sum(q_list$dof)

k <- sum(q_list$k)

tau2beta <- (qhet-dof)/k

################# Find Qnet, where Qnet = Y'BY #################

y <- unlist(all_data$y)

w <- solve(smat)

b <- w - w %*% X %*% solve(t(X) %*% w %*% X) %*% t(X) %*% w

qnet <- as.numeric(t(y) %*% b %*% y)

dof_qnet <- n-trts+1

constant1=sum(diag(b%*%p1))

constant2=sum(diag(b%*%p2))

################## Find untruncated tau2_omega #####################

tau2omega <- as.numeric((qnet-dof_qnet-tau2beta*constant1)/constant2)

# Find estimates and varcov matrix:

tau2beta_t <- max(0,tau2beta)

tau2omega_t <- max(0,tau2omega)

v <- smat + tau2beta_t*p1 + tau2omega_t*p2

v_inv <- solve(v)

Cov <- solve(t(X) %*% v_inv %*% X)

est <- Cov %*% t(X) %*% v_inv %*% y

################## Find I^2 statistics (Section 5.3) ##################

# Fit model under "random-effects and consistent" (RC) model (i.e. assuming tau2_omega is zero) (section 4.3):

tau2beta_con=(qnet-dof_qnet)/constant1

tau2_beta_contrun=max(0, tau2beta_con)

Vcon = smat + tau2_beta_contrun*p1

WWcon=solve(Vcon)

Cov_con=solve(t(X) %*% WWcon %*% X)

est_con=Cov_con %*% t(X) %*% WWcon %*% y

sd=diag(Cov)^0.5;

sd_con=diag(Cov_con)^0.5

p=pnorm(-abs(as.numeric(est))/sd)*2 ;

p_con=pnorm(-abs(as.numeric(est_con))/sd_con)*2

# Fit model under "common-effect and consistent" (CC) model (i.e. assuming tau2_beta and tau2_omega are zero):

Cov_CC_inv <- t(X) %*% w %*% X

Cov_CC <- solve(Cov_CC_inv)

power=1/(2*(trts-1))

# R and I statistics comparing RC model and CC model:

R12=det(Cov_con %*% Cov_CC_inv)^power

I_RC_CC=(R12^2-1)/R12^2

# R and I statistics comparing "random-effects and inconsistent" (RI) model and CC model:

R13=det(Cov%*%(Cov_CC_inv))^power

I_RI_CC=(R13^2-1)/R13^2

# R and I statistics comparing RI model and RC model:

R23=det(Cov%*%(solve(Cov_con)))^power

I_RI_RC=(R23^2-1)/R23^2

return(list(est=as.matrix(est), Cov=as.matrix(Cov), se=sd , p=p, tau2beta=tau2beta, tau2omega=tau2omega,

est_con=as.matrix(est_con), Cov_con=as.matrix(Cov_con),se_con=sd_con, p_con=p_con,

tau2beta_con=tau2beta_con, I12=I_RC_CC, I13=I_RI_CC, I23=I_RI_RC))

}

#OAK DATA

OAK=structure(list(d = c("BT", "BT", "BT", "BT", "AF", "AV", "BE",

"BE", "BE", "BE", "BE", "BE", "BE", "BV", "BV", "BV", "BV", "VI",

"HS", "BQ", "BJ", "BJ", "BJ", "AH", "AH", "AH", "AH", "AH", "AH",

"AH", "AH", "AH", "AH", "AH", "AH", "AH", "AH", "AG", "AG", "AG",

"AQ", "MD", "MD", "MD", "MD", "MD", "MD", "MD", "AD", "AD", "AD",

"AD", "AD", "AD", "BS", "BS", "BS", "BP", "BP", "BP", "AR", "CH",

"CH", "AK", "AK", "AK", "AL", "AL", "IH", "BH", "AE", "BI", "BU",

"VD", "BO", "AH", "ADH", "CVN", "ADM", "ADV", "GAL", "AHQ", "AGH",

"BIN", "COB", "BHV", "BDNV"), y = list(0.10789828, -0.18189296,

0.61413822, -0.82603693, -0.14916168, -0.11419721, -1.4097723,

-0.33240995, 0.06856271, -0.36464027, -0.75169228, -0.50324765,

0.49719666, -0.2426402, -2.93518003, -0.63975477, -0.77748203,

0.06109236, -1.19253525, -0.64570277, -0.8415101, 0.27921639,

0.14999677, -0.19771863, -0.46940718, -0.95646705, -0.39313575,

-0.20897436, -0.57880581, -0.02050818, -0.0678545, -0.44411973,

-0.43769753, -0.65908944, -0.45814044, -0.43840988, -0.20789905,

-0.51366242, -0.41349784, -4.28795429, -0.07497408, 0.05210705,

-1.06152028, -0.53068704, -0.06273315, -0.28286799, -1.77579377,

-0.77949258, -1.13556061, -0.75708101, 0.16615459, -1.69875885,

0.16551812, -1.06159269, -0.20099705, -0.37731101, -0.69964482,

0.35103883, -1.21532755, -0.07204235, 0.45557099, -2.44967895,

-0.71066998, -0.31747873, -0.3271303, -0.19472813, -0.72629004,

0.08966899, -0.80290605, -0.63473546, -1.01109974, -0.20138755,

-0.8227403, 0.15080312, -0.928263, -0.17013936, c(-0.233571462,

-0.025025514), c(-0.762857156, -0.433152233), c(-1.201925827,

-0.684690514), c(-1.071267074, -1.313976021), c(-0.053579736,

-0.177808846), c(-0.496204998, -0.267065054), c(-0.439873163,

-0.321458035), c(0, -0.689955396), c(-1.84964968, -0.19611917

), c(0.267938107, -0.171802395), c(-1.19775, -0.2603804,

-0.7811413)), s = list(0.0232674, 0.04839907, 0.06071628,

0.10877984, 0.03430561, 0.07154501, 0.09614721, 0.0780995,

0.09172009, 0.08170178, 0.15599594, 0.08253258, 0.1346979,

0.10898273, 0.27169231, 0.07841072, 0.06908316, 0.10004665,

0.0942214, 0.10213311, 0.14573089, 0.02595851, 0.06099994,

0.05911098, 0.07779299, 0.06190854, 0.02386021, 0.0565442,

0.19888567, 0.04349886, 0.20011511, 0.01123115, 0.1462782,

0.0767004, 0.07894128, 0.03310878, 0.04103685, 0.04493278,

0.13677825, 0.54134215, 0.06671351, 0.12921046, 0.05206926,

0.04705471, 0.0727871, 0.10100018, 0.26737741, 0.21519022,

0.06363698, 0.14288619, 0.10034509, 0.20238644, 0.11804995,

0.01334994, 0.07862088, 0.21767532, 0.23581952, 0.0760269,

0.10389677, 0.10006488, 0.12110769, 0.35594513, 0.05061601,

0.02227884, 0.1023655, 0.09854855, 0.05777662, 0.01066845,

0.08644658, 0.11056433, 0.05638952, 0.07885916, 0.1034265,

0.28652648, 0.17041677, 0.17374879, structure(c(0.049754964,

0.016393443, 0.016393443, 0.033062697), .Dim = c(2L, 2L)),

structure(c(0.284852637, 0.083333333, 0.083333333, 0.263094018

), .Dim = c(2L, 2L)), structure(c(0.040793869, 0.014925373,

0.014925373, 0.045750321), .Dim = c(2L, 2L)), structure(c(0.563756286,

0.166666667, 0.166666667, 0.595918499), .Dim = c(2L, 2L)),

structure(c(0.037832677, 0.0125, 0.0125, 0.037324696), .Dim = c(2L,

2L)), structure(c(0.251905638, 0.066666667, 0.066666667,

0.134522062), .Dim = c(2L, 2L)), structure(c(0.024831019,

0.007874016, 0.007874016, 0.024362932), .Dim = c(2L, 2L)),

structure(c(0.212669683, 0.076923077, 0.076923077, 0.231583482

), .Dim = c(2L, 2L)), structure(c(0.350038469, 0.142857143,

0.142857143, 0.412386578), .Dim = c(2L, 2L)), structure(c(0.193227337,

0.0625, 0.0625, 0.188114918), .Dim = c(2L, 2L)), structure(c(0.18278513,

0.04, 0.04, 0.04, 0.16223164, 0.04, 0.04, 0.04, 0.1703878

), .Dim = c(3L, 3L)))), .Names = c("d", "y", "s"))

fn(OAK)
